# Thermal imaging and LiDAR mapping for disaster management with hexacopter

**DOI:** 10.1016/j.ohx.2026.e00841

**Published:** 2026-09-24

**Authors:** Harshitha Vuucha, Jeff Jomon, Dheeraj Somasundaram, Monica Subashini M.

**Affiliations:** Vellore Institute of Technology (VIT), Vellore Campus, Tiruvalam Road Katpadi, Vellore 632014, Tamil Nadu, India

**Keywords:** LiDAR, Lepton 3.5 thermal camera, Disaster Risk Reduction (DRR), Remote-controlled aircraft

## Abstract

Natural disasters such as floods and tsunamis continue to pose significant threats, a growing population coupled with inadequate preparedness greatly worsening their impact on human life and infrastructure. This growing danger highlights the critical need for Disaster Risk Reduction (DRR), which is in line with international objectives that rely on DRR for success, including the Biodiversity Agenda, the New Urban Agenda, the Paris Climate Agreement, and the Sustainable Development Goals. Because of this, a sophisticated aerial system for quick disaster response was proposed in this paper. It makes use of a remote-controlled aircraft that is equipped with modern technologies like LiDAR for mapping and a Lepton 3.5-based thermal camera for detecting lifeforms. The combination of these technologies with dynamic moving parts allows for improved navigational accuracy. Our prototype is successfully able to detect hotspots and potential hazards using cost-effective components, including a modified ESP32 and a Lepton 3.5 thermal camera. Although the LiDAR system effectively demonstrated 3D terrain mapping through point cloud generation, the integration with the hexacopter platform was unfortunately constrained by power limitations. Regardless, it is safe to suggest, even with just the Lepton based thermography, responders would have considerably better situational awareness, reduced response times, and overall effective disaster management.

## Graphical abstract

**Figure:**
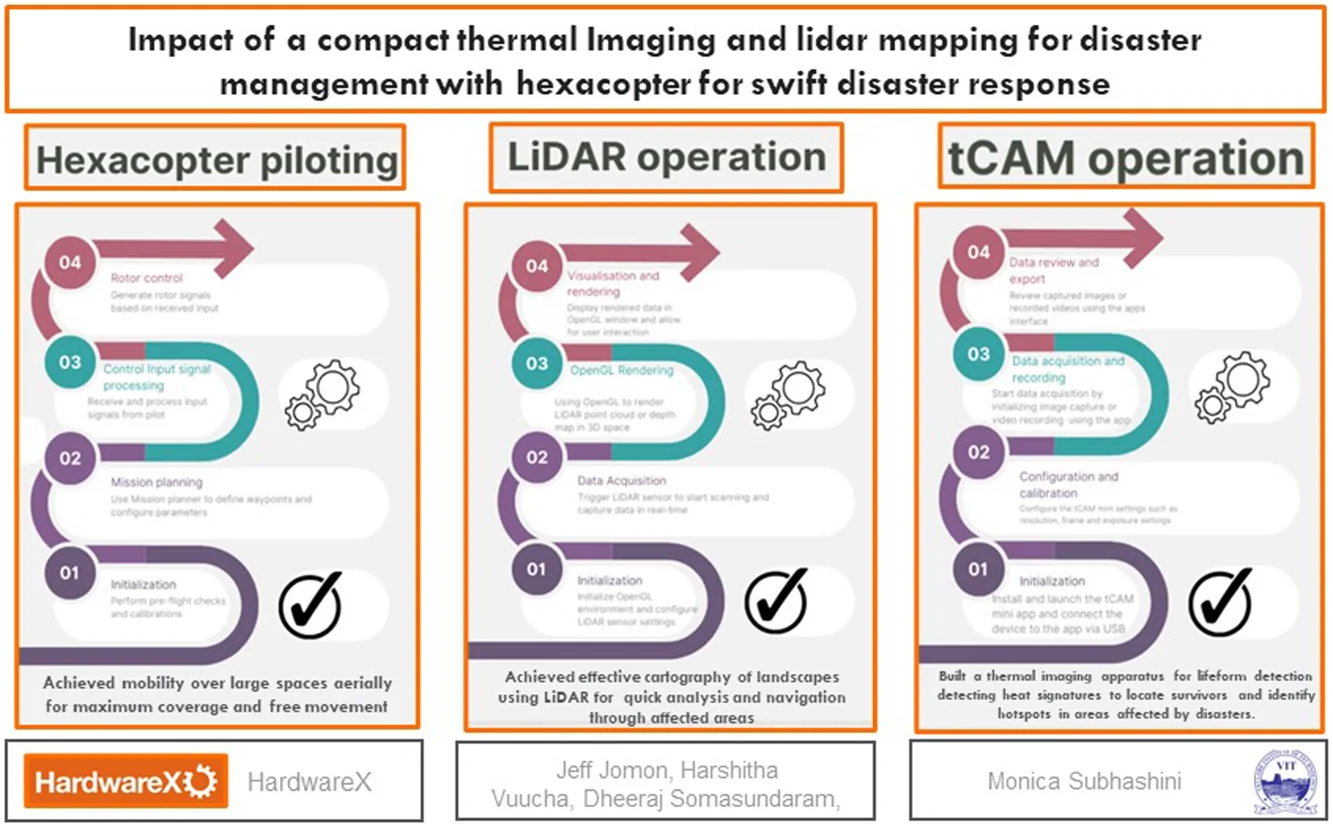

**Table:** 

**Specifications table**	

Hardware name	Rapid Response

Subject area	• Embedded Electronics and Robotics

Hardware type	• Terrain Surveillance and Disaster Management • Risk Reduction

Closest commercial analog	DJI Drone Variants - MAVIC, MAVIC PRO etc.

Open source license	GNU GENERAL PUBLIC LICENSE Version 3, 29 June 2007 For tCam Mini

Cost of hardware	Rs. 73689.35 **(Bill of materials).**

Source file repository	https://doi.org/10.5281/zenodo.11127608https://doi.org/10.5281/zenodo.11127733https://doi.org/10.5281/zenodo.11127759https://doi.org/10.5281/zenodo.11127784

## 1 Hardware in context

To manufacture the hardware equipment for the hexacopter, the chassis was assembled and then six propellers, which were powered by brushless DC motors, were attached to it. Pilot testing and initial flight calibrations were performed, after which the final flight and field testing of the product were executed. The electronic component for the Lepton 3.5 thermal camera was connected using SPI communication protocols. Although we were able to get comprehensive coverage the LiDAR scanner. We were unable to incorporate the system during flight test,due to significant payload capacity and power limitation, which remain common constraints for compact UAV-based LiDAR systems [1], [2].

Real-time wireless transmission tests were conducted to analyze and evaluate the performance of the thermal life-form detection system such as the maximum achievable frame rate. For flight operation, the Lepton sensor was enclosed in a 3D-printed housing to minimize stress and vibration, and the mounting assembly was completed accordingly. Subsequently, flight tests were performed to validate the hexacopter system and assess its overall operational performance.

In terms of the software, the LiDAR apparatus was principally programmed with Embedded C/C++ code. This enabled the user to have access to the 3D Point Cloud system through an interface that was designed using OpenGL, consistent with established LiDAR visualization approaches [3], [4], [5], [6]. For the purpose of interface creation, the Xojo application development platform was utilized, which was implemented using Python command lines. The open-source tool Mission Planner, which is compatible with APM2.8 and Pixhawk flight controllers, was used to verify and calibrate the hexacopter.

## 2 Hardware description

Compared to other technologies, the integration of LiDAR and the Lepton 3.5 thermal camera on a hexacopter has the potential to provide improved precision, mobility, and situational awareness for disaster response applications [1], [2], [3], [7], [8], [9], [10], [11].

Hexacopters often have lower operating expenses than other larger aerial platforms, making them an affordable choice for frequent reconnaissance and monitoring missions during disaster response [2], [9], [10]. They provide redundancy with many rotors, lowering the risk of failure and improving overall flight safety, which is critical for missions in demanding and hazardous settings, as well as allowing for fast deployment and operation in emergencies without requiring considerable setup or infrastructure [6], [9], [10], [12], [13], [14], [15]. Furthermore, their exceptional maneuverability also allows them to navigate confined spaces and difficult terrain, providing access to isolated or otherwise inaccessible disaster zones [8], [9], [10], [12], [13], [14], [15]

It also has a significant benefit over other aerial crafts, such as quadcopters, in that it can continue to fly if one propeller breaks, which is a possible occurrence in crisis management and an essential precaution [2].

The Garmin LiDAR Lite V3 is unique in its compact size, lightweight design, and high-performance capabilities. Its ability to produce accurate distance measurements while maintaining a wide field of vision makes it an excellent choice for disaster management, terrain mapping, and object detection activities [1], [3], [4], [5], [6].

By utilizing the Lepton 3.5 thermal camera to provide simultaneous high-resolution anomaly detections, this system outperforms standard disaster management methods by expediting operations with comprehensive, real-time data viewing and analysis capabilities [7], [8], [11], [16].

The Lepton 3.5 thermal camera has transformed thermal imaging applications due to its tiny size and great sensitivity. It can be easily integrated into a wide range of devices, including smartphones, drones, and IoT devices. It also performs more effectively, allowing for accurate temperature monitoring and thermal anomaly identification even in difficult situations, thanks to improved thermal sensitivity and image quality with lower power consumption [7], [11], [16]. Because this technology is fairly inexpensive and simple to use, thermal capabilities are now widely available to a larger number of clients for a wider range of applications [7], [16].

## 3 Design files summary

Using the Garmin Lite V3 and Lepton 3.5 thermal camera, it is possible to construct a radiometric thermal imaging equipment and LiDAR 3D point cloud system [1], [2], [3], [12], [13], [14]. The following links provided below contain all of the important applications and instructions that are needed to replicate this project.

Among the files that are given for the LiDAR are the hardware schematic, which illustrates the connections that need to be made, as well as the Arduino files for the Garmin Lite V3 and the HS-422 servo motor’s pan and tilt movement.

There are three distinct files that make up the thermal imaging apparatus. One of these files is for the 3D printing that was done on Simplify3D. There are four STL files, and one print of each of them must be taken. Additionally, there are two prints of the vertical holder. Following this is the programming folder, which is for updating the firmware of the Lepton 3.5 and ESP32-WROVER-based breakout board. The instructions for this can be found in the zip file. Finally there is the main tCam Mini file, which not only includes the program that can be used on the desktop to see the thermal feed but also the firmware that needs to be loaded onto the Lepton 3.5 and ESP32-WROVER-based breakout board to complete the thermal system.

All necessary instructions and design files are available in the Zenodo repository (See Table 1).

*1. Included is the code necessary to generate a 3D point cloud via an OpenGL application, along with the code for the HS-422 servo’s pan and tilt. Furthermore, a schematic depicting the essential hardware connections is attached.*

**Table 1:** Summary of design files.

Design filename	File type	Open source license	Location of the file
1. LiDAR 3D Point Cloud System using a Garmin Lite V3	OpenGL Application, Arduino code and Schematic PNG	Creative Commons Attribution 4.0 International	https://doi.org/10.5281/zenodo.11127608

2. Programmer Files for Firmware	serial updater application EXE file	GNU GENERAL PUBLIC LICENSE	https://doi.org/10.5281/zenodo.11127733

3. STL Files for 3D Enclosure	4 STL files	GNU GENERAL PUBLIC LICENSE	https://doi.org/10.5281/zenodo.11127759

4. tCam Mini Main Firmware using the Lepton 3.5 Camera	2 ZIP Files	GNU GENERAL PUBLIC LICENSE	https://doi.org/10.5281/zenodo.11127784

*2. tCam Mini Firmware upload — for Programming and additionally two prints of tCam Mini gp mount v file must be printed along with all other STL Files.*

*3. These contain the STL files for 3D printing to duplicate the enclosure for the tCam Mini*

*4. This holds the main open-source firmware developed by Dan Julio that utilizes the Lepton 3.5 thermal camera for radiometric thermography as well as the Desktop Application created for the same.*

## 4 Bill of materials summary

The complete list of materials and components required for the proposed system is provided in Table 2.

*The total cost of all components is Rs. 73,689.35 (INR)*

**Table 2:** Bill of materials summary.

Component	Supplier/ Source	Number	Cost per unit (INR)	Total cost (INR)
F450 F550 Frame Landing Gear Landing Skid	Robu.in	1	Rs. 383	Rs. 383

DC JACK-005A	Robu.in	1	Rs. 5	Rs. 5

SafeConnect XT-60 Connector Harness for Parallel Connection Battery Adapter Cable (2M-1F)	Robu.in	1	Rs. 169	Rs. 169

Readytosky 30cm Nylon Strap Belt for RC Lipo Battery	Flyrobu.in	1	Rs. 69	Rs. 69

1045 Propeller 10in 10 × 4.5 For Drone	Flyrobu.in	7	Rs. 85	Rs. 595

Anti-Vibration Shock Absorber for APM/KK/MWC/PixHawk! Shock Absorber	Flyrobu.in	1	Rs. 360	Rs. 360

APM/Pixhawk Power Module V6.0 Output BEC 3A XT60 Plug 28V 90A	Robu.in	1	Rs. 699	Rs. 699

Flysky FS-i6X 2.4GHz 6CH AFHDS 2A RC Transmitter With FS-iA10B 2.4GHz 10CH Receiver	Robu.in	1	Rs. 5399	Rs. 5399

A2212 1000 KV BLDC Brushless DC Motor for Drone (Soldered Connector	Robu.in	6	Rs. 421	Rs. 2526

F550 Hexa-Copter Frame, Landing Gears and Integrated PCB Kit	Robu.in	1	Rs. 2099	Rs. 2099

APM 2.8 Flight Controller with Built-in Compass	Robu.in	1	Rs. 6649	Rs. 6649

Standard 30A BLDC ESC Electronic Speed Controller with Connector	Robu.in	6	Rs. 519	Rs. 3114

Garmin LiDAR Lite V3	tanotis.com	1	Rs. 23215	Rs. 23215

HS-422 Servo	Robu.in	2	Rs. 1666	Rs. 3332

Lynxmotion Pan and Tilt Kit/Aluminium	robotshop.com	1	Rs. 3752.55	Rs. 3752.55

FLIR Lepton 3.5	groupgets.com	1	Rs. 14164.19	Rs. 14164.19

tCam-Mini rev4 - (onboard antenna) Wireless streaming thermal camera board	groupgets.com	1	Rs. 7158.61	Rs. 7158.61

## 5 Build instructions

### 5.1 Setting up the LiDAR imaging setup

**Step 1:** For the hardware component you will need: A LiDAR sensor for distance measurement, two HS-422 servo motors for pan and tilt motion. and an Arduino Uno microcontroller for controlling the servos and acquiring sensor data.

**Step 2:** The LiDAR sensor is connected to the Arduino Uno through several pins The LiDAR sensor pin configuration is illustrated in Fig. 1.

• Pin 1 (Red wire): Connected to the 5 V DC supply.
• Pin 2 (Orange wire): Connected to Arduino digital pin 4 and used as the sensor enable pin.
• Pin 3 (Yellow wire): Used as the mode control pin. It is connected through two parallel 2.2 kiloohm resistors, with one end connected to Arduino digital pin 2 and the other to PWM pin 3 for bus contention control.
• Pin 4 (Green wire): I2C clock (SCL) line connected to Arduino A5/SCL.
• Pin 5 (Blue wire): I2C data (SDA) line connected to Arduino A4/SDA.
• Pin 6 (Black wire): Connected to system ground.

**Step 3:** Next, the two servo motors are connected to the Arduino Uno:

**Fig. 1:**
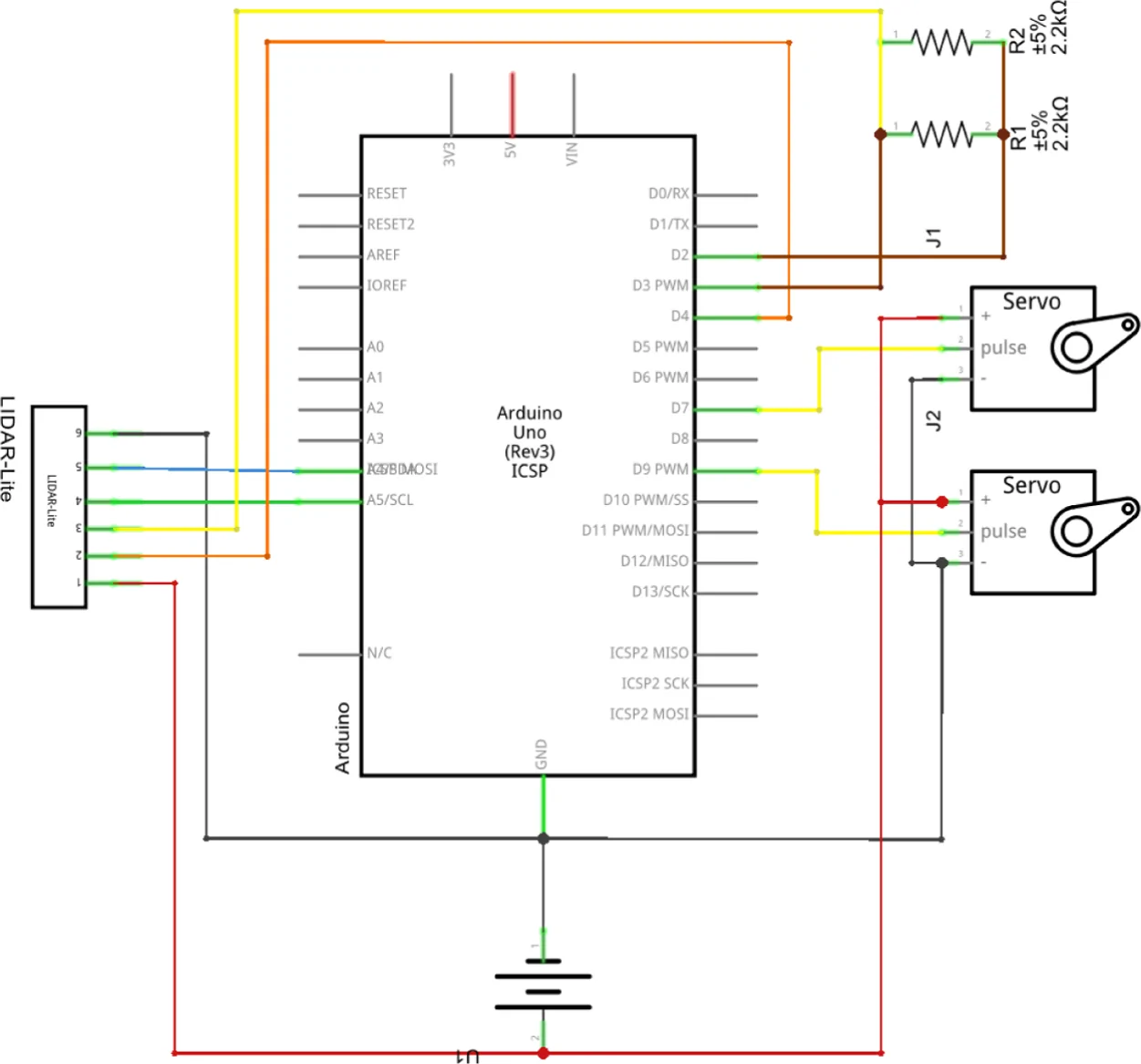
LiDAR Pin Configuration.

• Pan Servo: Signal wire connected to PWM digital pin 9; power and ground connected to 5 V and GND respectively
• Tilt Servo: Signal wire connected to digital pin 7; power and ground connected to 5 V and GND respectively.

Written in C++ and OpenGL, the computer interface for the prototype is open source. It receives data from the Arduino, analyzes it, converts it to Cartesian coordinates, and displays the results on the screen. Every point is represented on the screen as a cube, and its color changes based on angles and distance. Additionally, the interface is in charge of communicating with the Arduino and sending a beginning code that instructs the prototype to begin scanning. While the distances are being read and relayed, every scan is displayed in real time on the screen. While the scanning is going on, you can move around in the 3D view. The program enables the scan to be saved as a file and then loaded at a later time. The complete LiDAR system, including the Arduino Uno, servo motors, and sensor integration, is illustrated in Fig. 2.

**Fig. 2:**
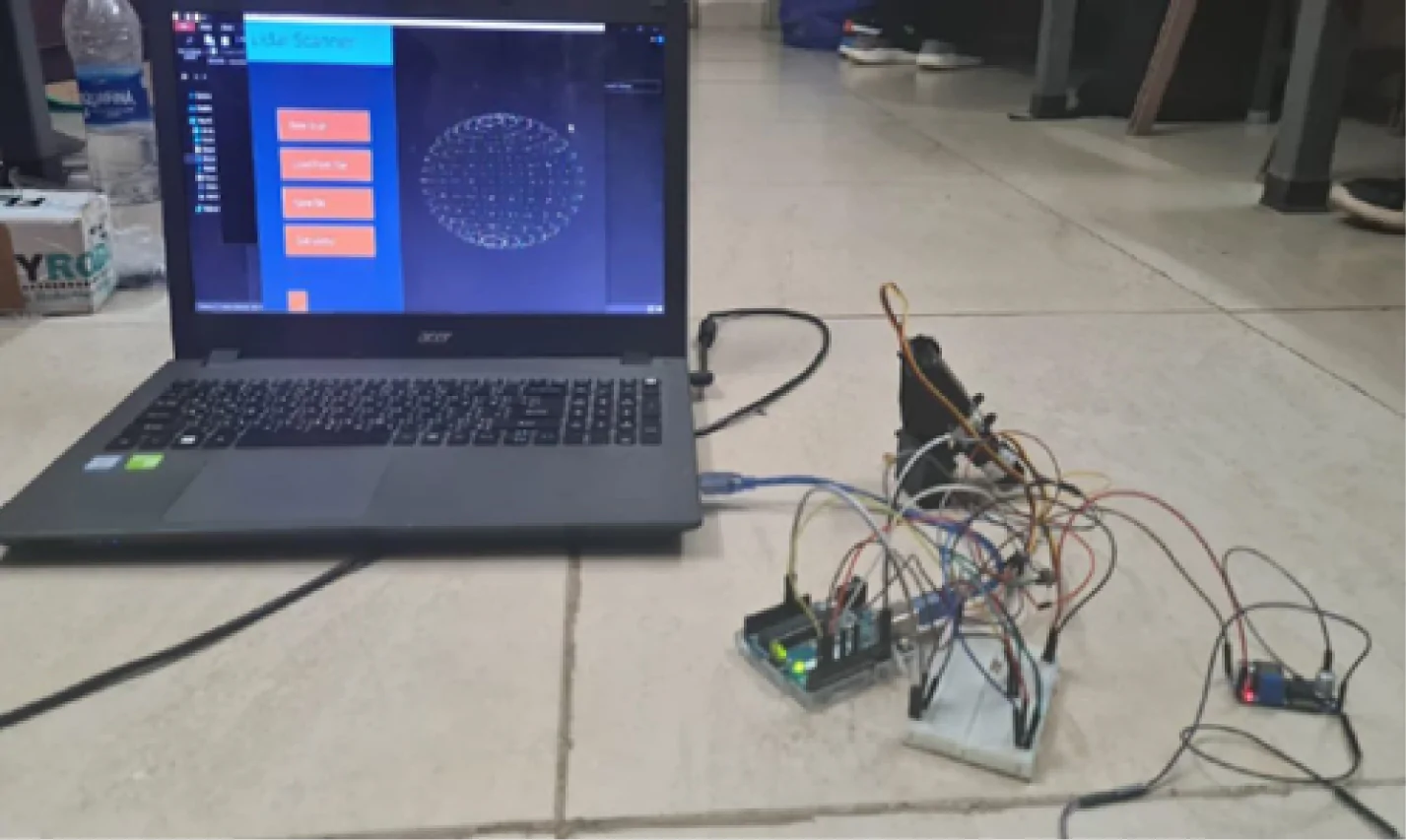
LiDAR system.

### 5.2 Setting up pan and tilt setup for LiDAR

**Step 1:** The Pan and tilt assembly begins by attaching the multi-purpose servo bracket to one servo, followed by attaching the ”C” servo bracket to the multi-purpose bracket and securing it with a nylon acorn nut.

**Step 2:** The servo is then installed into the multi-purpose bracket using push-in fasteners, and the ”C” bracket is attached to the servo. Finally, the LiDAR is affixed to the pan and tilt assembly with double-sided tape.

The pan-and-tilt assembly for orienting the LiDAR sensor is presented in Fig. 3

**Fig. 3:**
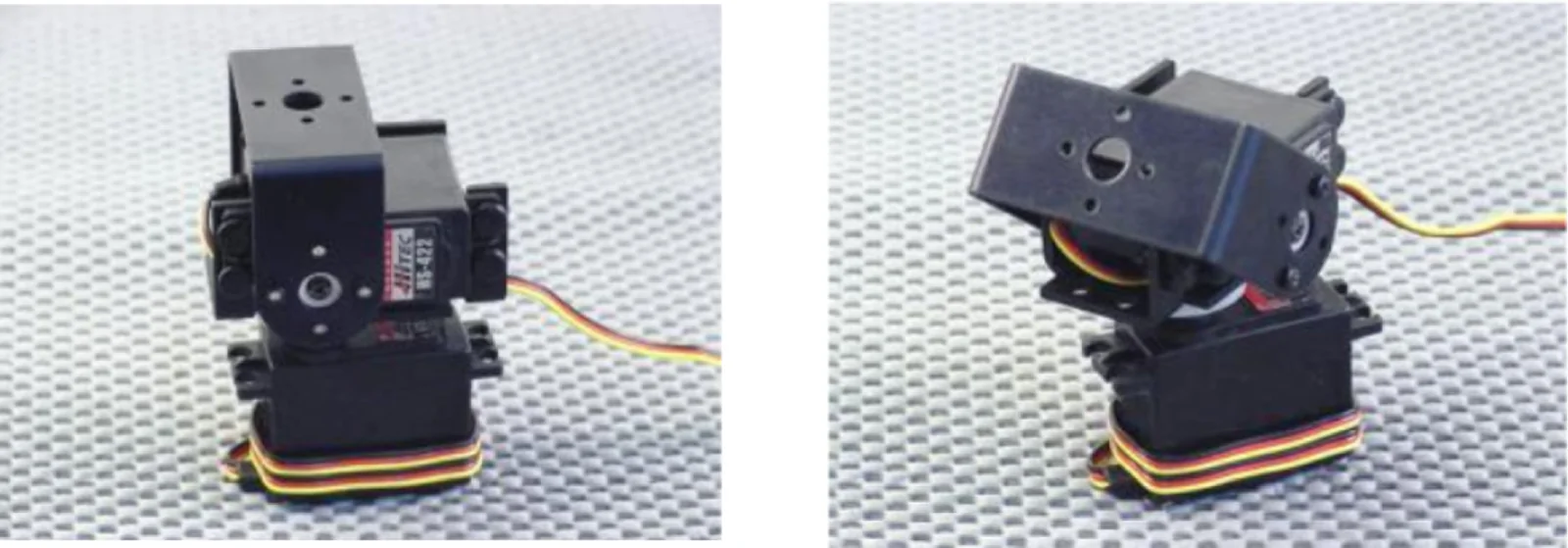
Pan and Tilt Assembly System.

### 5.3 Building of the hexacopter

The assembly process of the hexacopter chassis is illustrated in Fig. 4.

**Step 1:** The motors were first mounted to the arms of the device using M3 screws. Bullet connectors were fed through the frame first to ensure proper positioning of the Electronic Speed Controllers.

The installation of the A2212 brushless motors onto the hexacopter frame is shown in Fig. 5.

**Step 2:** The power distribution board was then positioned on the bottom frame piece and the wiring was completed after which,we move to the soldering was then done, with pads on the power distribution board and ESCs being tinned with solder. Flux was used to aid in solder flow and prevent cold solder joints. Wires were soldered to the appropriate pads on the board and ESCs.

**Fig. 4:**
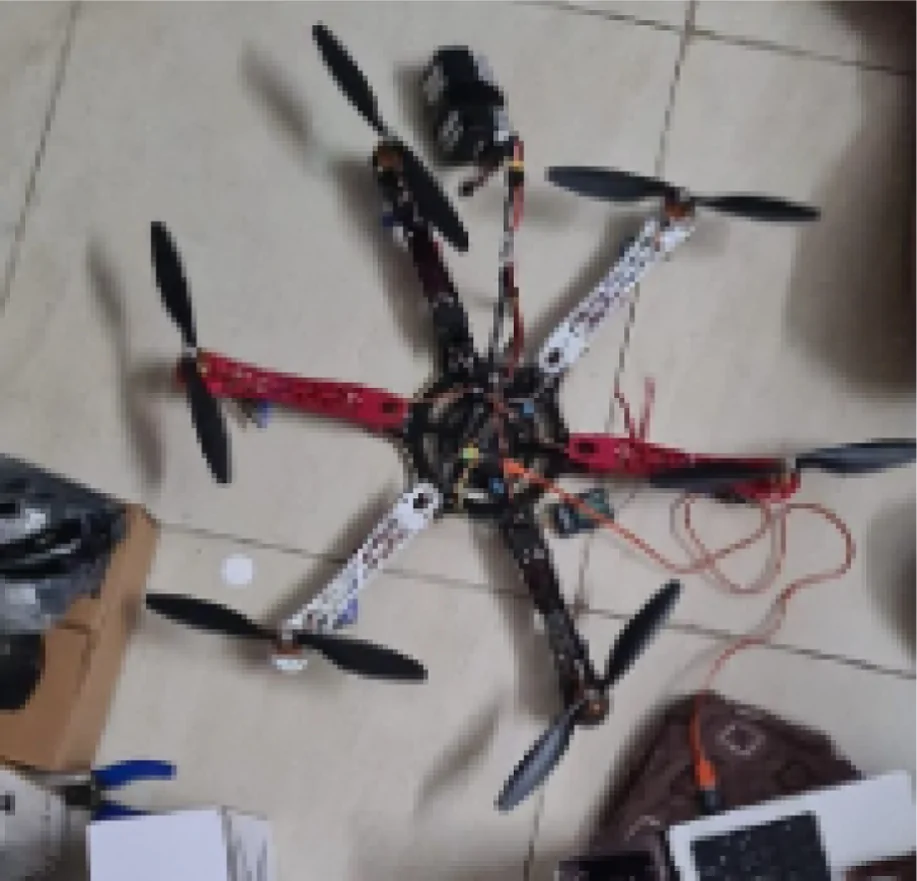
Building the chassis of the hexacopter.

**Fig. 5:**
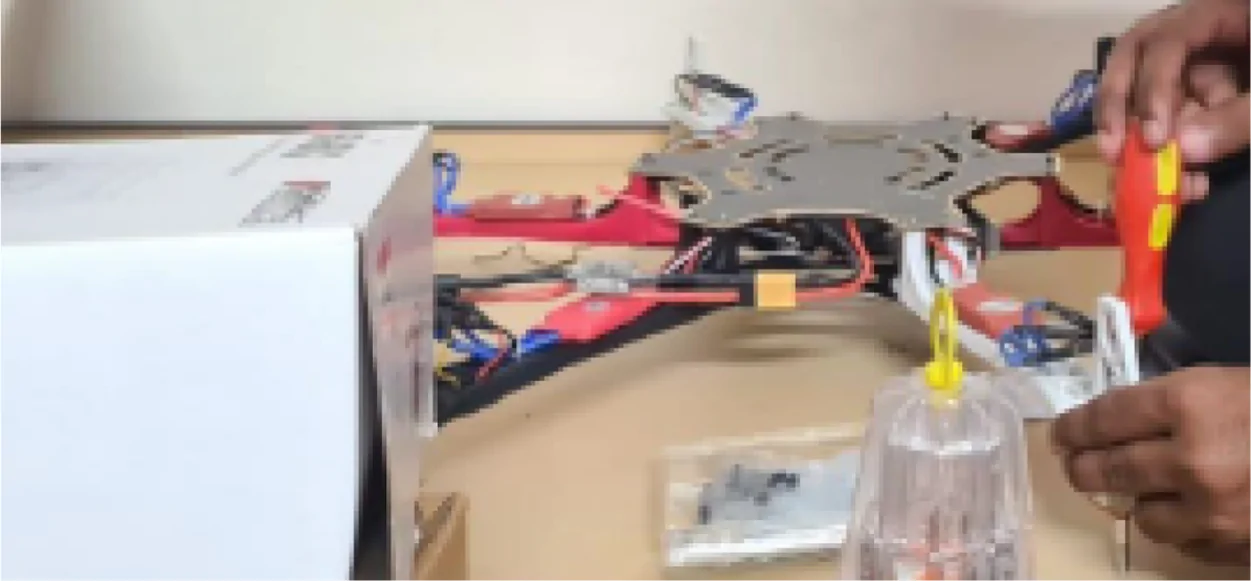
Bolting the A2212 motors in place.

**Step 3:** The top plate of the device was finally installed, enclosing the electronics and providing structural support, and care was taken to ensure that the battery was positioned correctly along the center section. The layout this specific hexacopter design had was a Hexa-X motor layout.

The complete hexacopter configuration is presented in Fig. 6.

**Step 4:** With regards to this layout, we connect the signal pin of each motor to the numbered output channels of the controller. Moving on, we configure the clockwise and anticlockwise movement of each motor with the other two pins of the motors. The first four channels of the receiver are connected to the controller for drone control and two more channels are connected to access the flight mode as well. The pin on the top end of the receiver is the signal pin, then the VCC pin, and finally the GND pin.

**Fig. 6:**
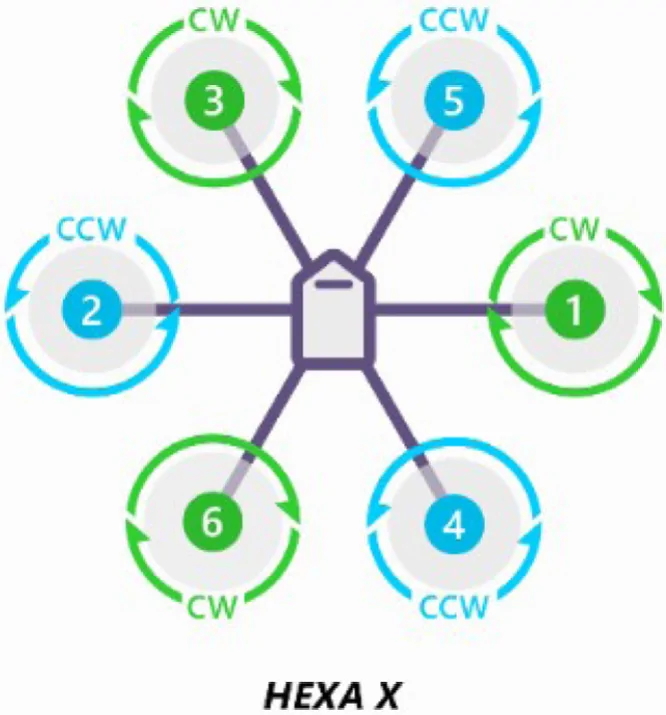
Hexacopter Configuration.

The receiver-to-flight-controller wiring configuration is illustrated in Fig. 7, showing the pin assignments used for signal, power, and ground connections. The final assembly of the hexacopter prior to installing the landing gear is shown in Fig. 8.

**Step 5:** The respective connections are made from the receiver to the controller with the first channel having all three pins connected to power the receiver and the remaining five channels being connected to signal pins.

**Fig. 7:**
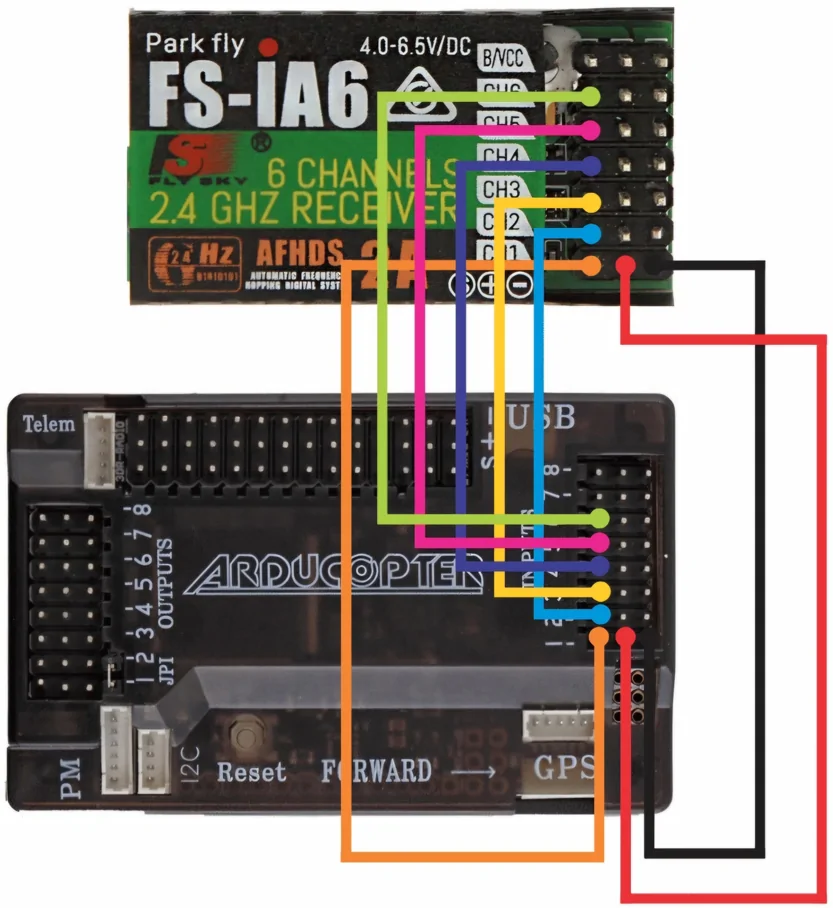
Receiver to Flight Controller Pin Configuration.

**Fig. 8:**
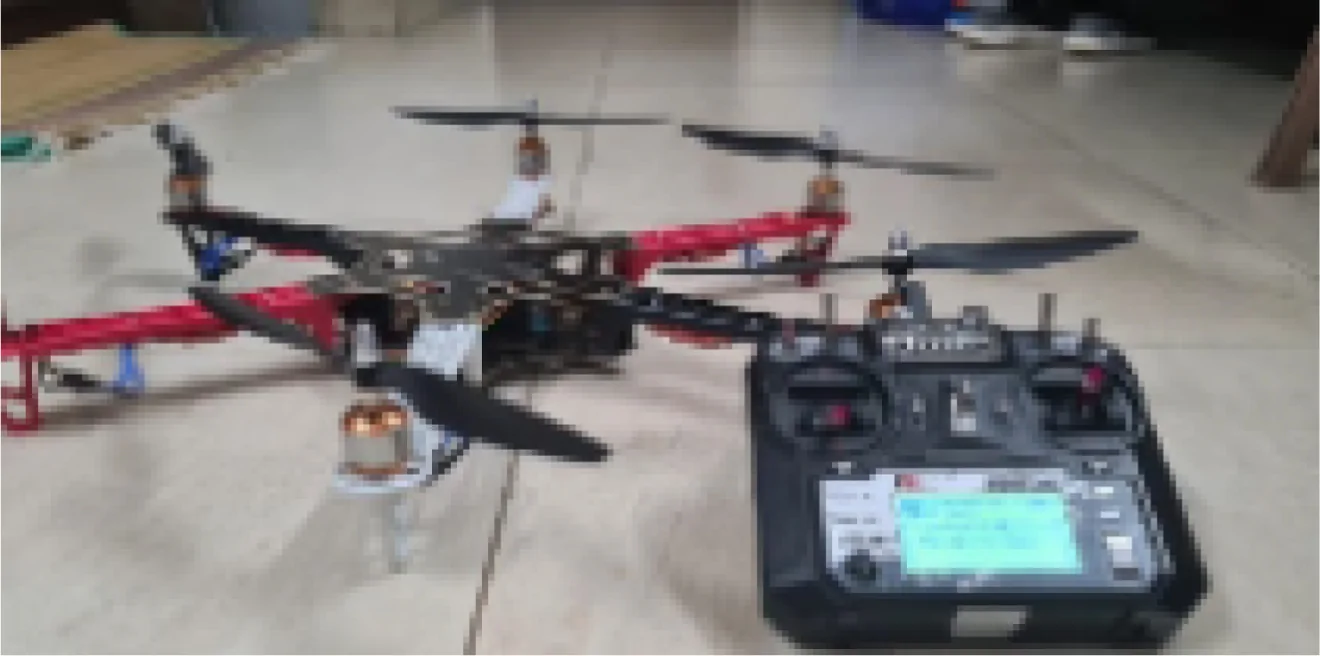
Final Assembly of the hexacopter without the landing gear.

### 5.4 Configuring mission planner

Mission Planner is an open-source application which enables virtual ground control for copters, planes, or rovers. It configures multiple settings for autonomous devices, ensuring efficient performance.

**Step 1:** To program the APM 2.8 Flight Controller, it is connected to a PC and a proper COM port is selected with a baud rate of 115200. Under the Initial Setup tab, the Install Firmware tab is chosen, the setup type is selected (e.g., Hexa X), and the firmware is booted to the board. Finally, the connect option is selected to establish the MavLink connection between the APM2.8 and the PC.

**Step 2:** For calibration of the Compass and Accelerometer. First select the Hexa X frame type for the hexacopter (See Fig. 9) under the Initial Setup tab. Move hexacopter until all the red dots disappear. Finally, the calibration information is automatically set.

The calibration procedures for the accelerometer and compass performed in the Mission Planner software are illustrated in Fig. 10, Fig. 11, respectively.

**Step 3:** For connection and calibration of the Radio Control, the receiver is connected to the APM Flight Controller, the GND, positive supply and signal pins are matched. A red light on the Receiver indicates a successful connection. The receiver with the Transmitter is bound and then next is the radio calibration section. The sticks, knobs, and switches are moved to their extremes to calibrate the parameters and the settings are saved by clicking ”click when done”.

**Fig. 9:**
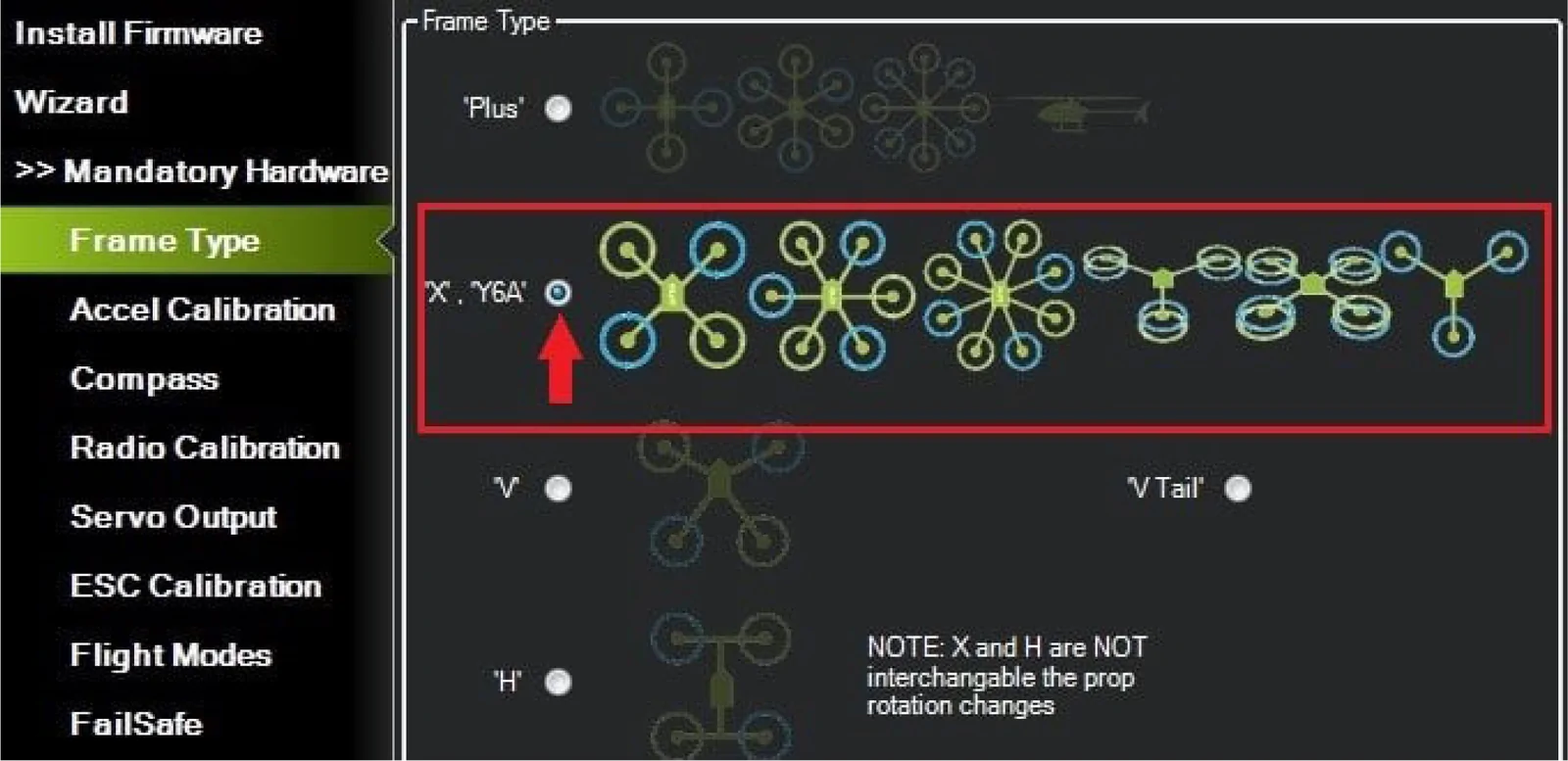
Mission Planner: Frame Type Selection.

**Fig. 10:**
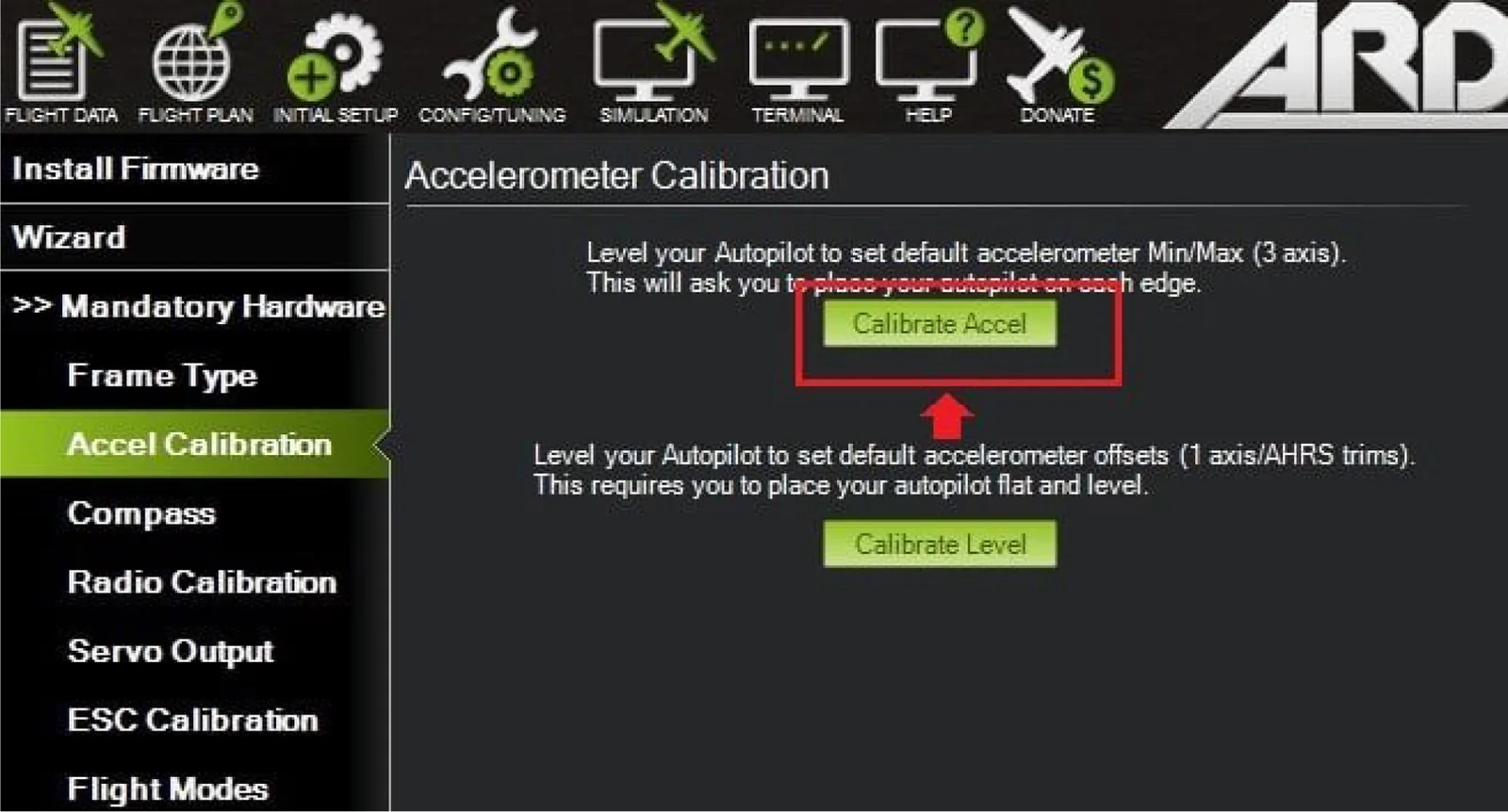
Calibration of Accelerometer.

**Fig. 11:**
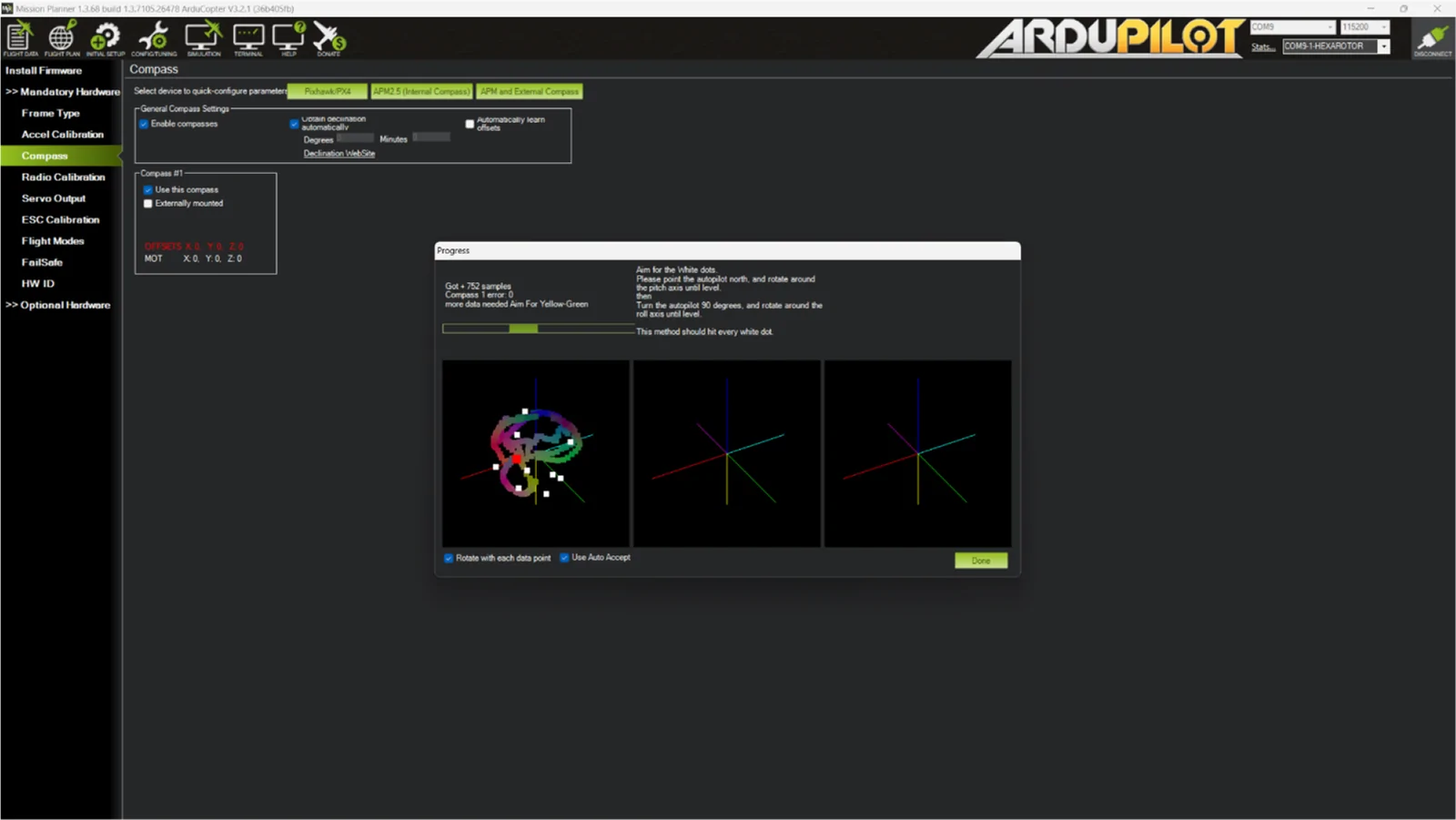
Calibration of Compass.

The radio calibration interface and channel calibration process are shown in Fig. 12

**Step 4:** To program ESCs, the transmitter is turned on, the throttle stick is pulled to the top, and then two beeps will be heard. The throttle stick is held at the top for another two seconds until a tune indicates entry into programming mode. Program parameters are selected by holding the throttle stick at the top and pulling it to the bottom within two seconds of the corresponding sound. The values are chosen and saved and then repeated for further programming. To exit, the throttle stick is pulled to the bottom and held until a tune again indicates completion.

**Fig. 12:**
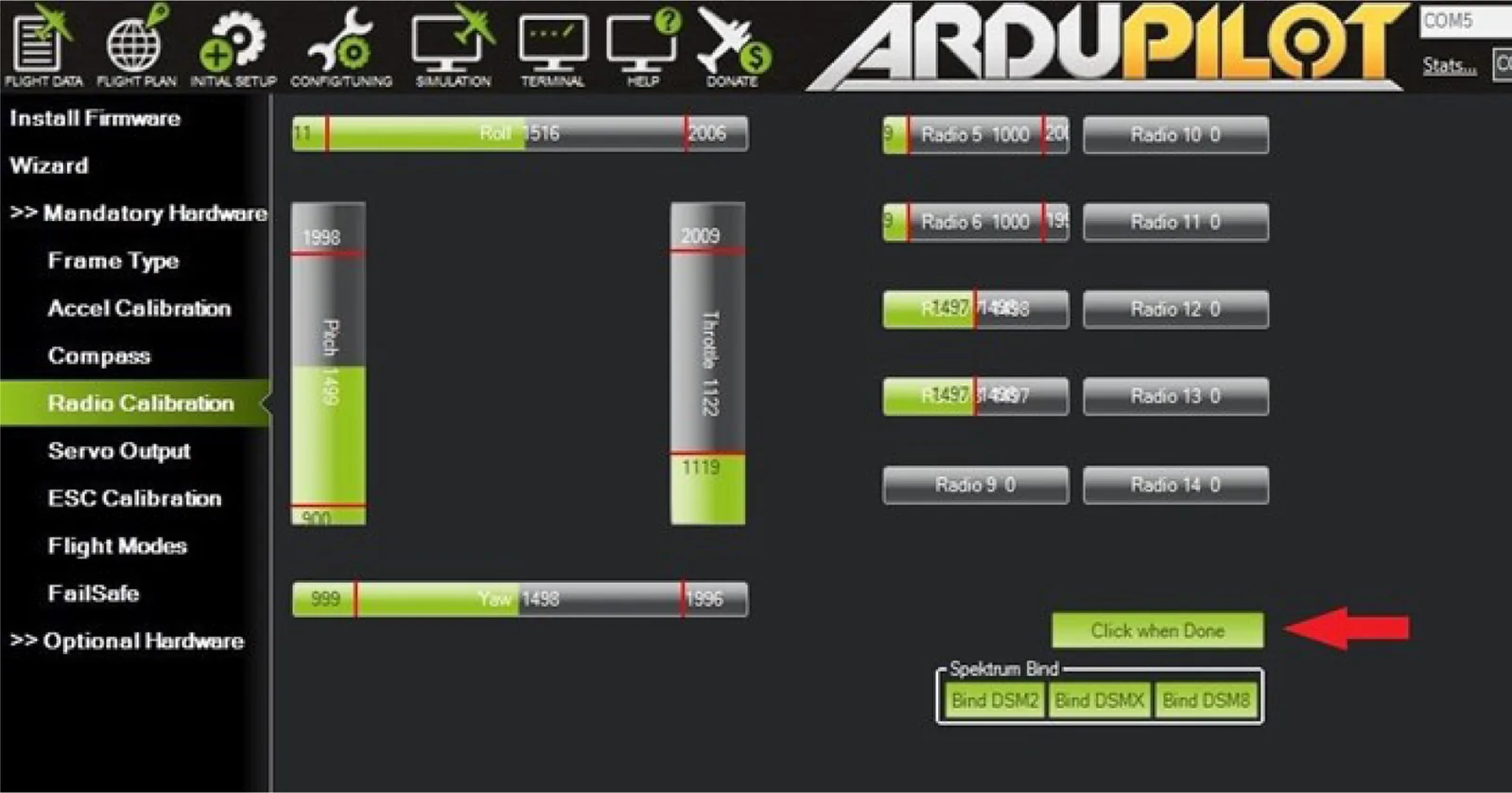
Calibration of Radio.

### 5.5 Setting up the ESP-32 based thermal imaging setup

tCam-Mini is an open-source hardware and software. It is designed to make radiometric data from a Lepton 3.1R and 3.5 thermal imaging modules available via Wifi for remote access.

**Step 1:** Download and unzip the Espressif utility program somewhere on your PC, next you also want to download and store the precompiled firmware binaries files. There are three files that make up a complete set of firmware to program: bootloader, partitions singleappd and tCamdot.

**Step 2:** Make sure your computer recognizes the camera as a serial port (COM port) by connecting the board to it using a USB cable. In the utility application, choose that COM port.

**Step 3:** Start the Espressif utility application by running the flash download tools v3 program. Choose the ESP32 Download tool from the options it offers. Load the three firmware files and configure the programming settings, baud rate, and serial port.

The firmware upload procedure for the flight controller is presented in Fig. 13.

**Step 4:** After this is completed (approximately 10–20 s) and the utility program displays the word “FINISH”, you can restart the camera by pushing the reset button on the board or unplugging and reconnecting the USB power cable. The dual-color LED shows useful status. If everything goes well, you should see it briefly turn red, then gently blink to yellow. This shows that the camera has properly booted and is waiting for a computer to connect to its wifi.

**Fig. 13:**
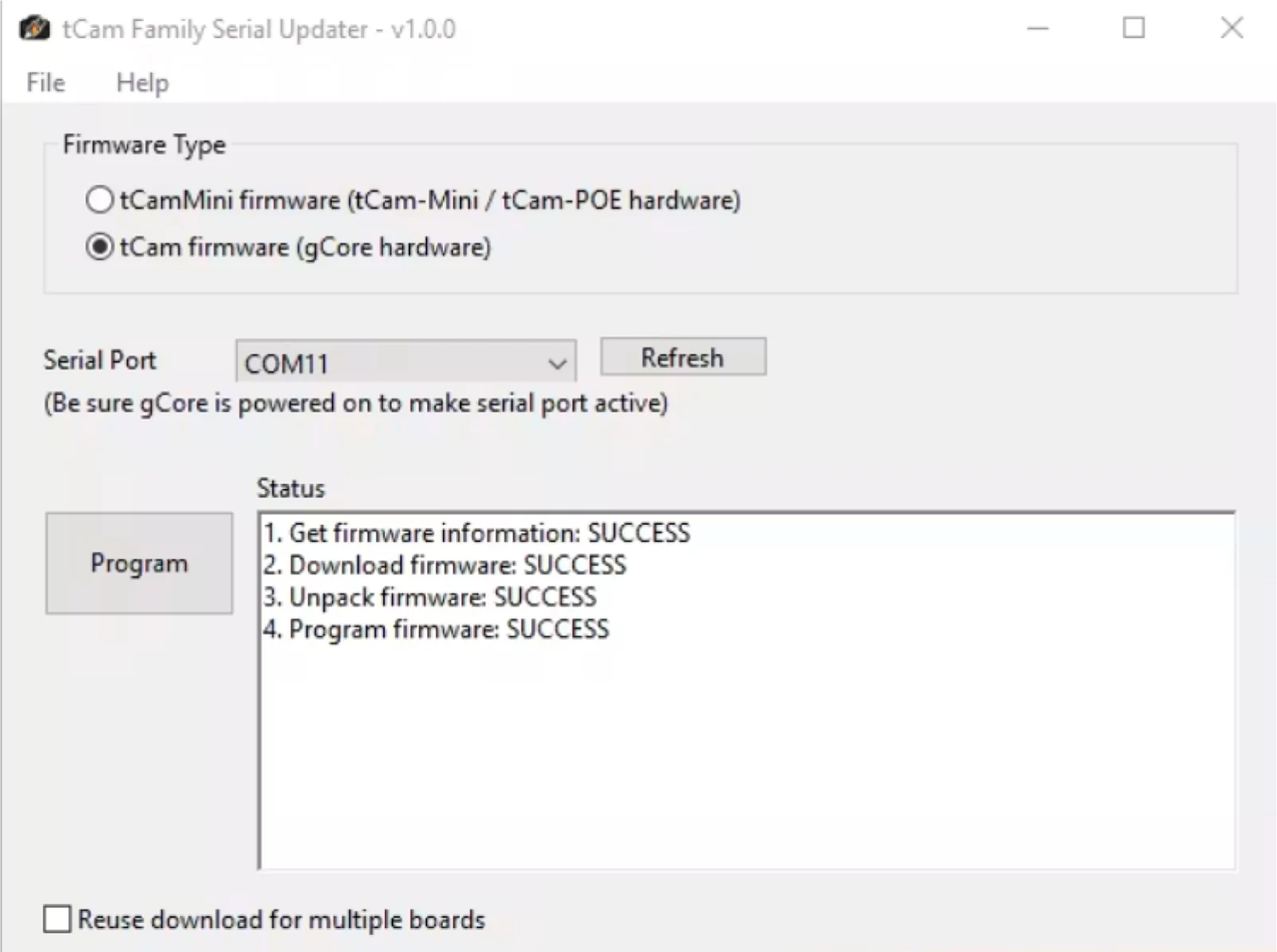
Firmware Upload.

The process of uploading firmware to the ESP32-WROVER breakout board is illustrated in Fig. 14.

**Step 5:** The tCam-Mini will function as a home router-like WiFi access point (AP) and advertise an SSID in the format “tCam-Mini-XXXX”, where “XXXX” is a string of four hexadecimal characters. To communicate with tCam-Mini, your computer must be connected to this WiFi network. Before you disconnect from your current home network, make sure you have downloaded, unzipped, and installed the companion desktop application.

**Fig. 14:**
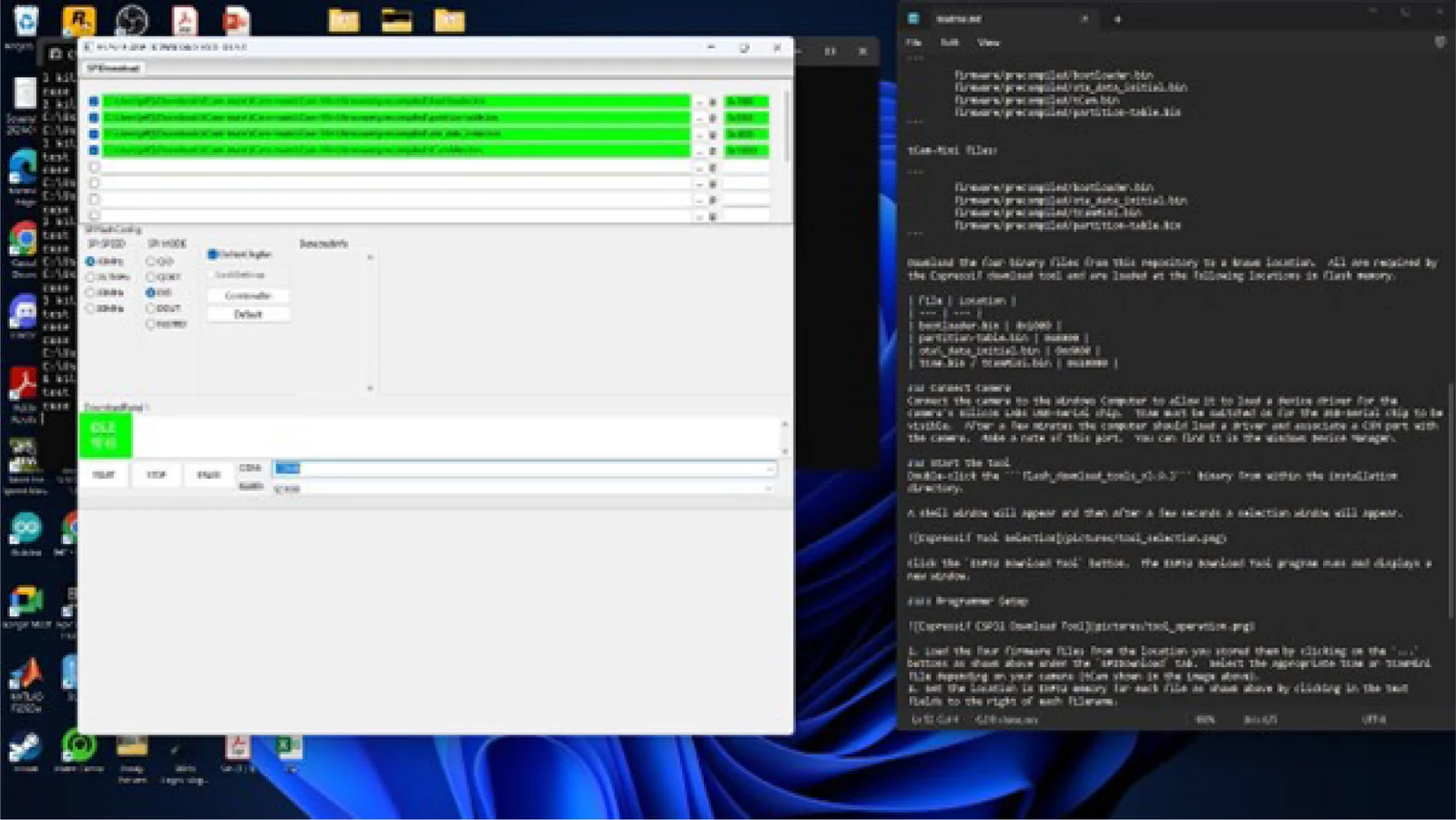
Uploading Firmware into the ESP32 Wrover Breakout Board.

**Step 6:** First, connect your PC’s WiFi to the tCam-Mini. Launch the software after that. Choose the Preferences button from the application’s toolbar. The camera’s default IP address is “192.168.4.1”. Select this and press the “Connect” button. An update indicating a connection to your camera should appear on the status line.

### 5.6 3D printing

The 3D printing process for the tCam-Mini casing involves several steps. In most cases, the casing design is exported to an STL file before being imported into a slicing application like Simplify3D. Individually, each component of the casing is utilized within the application, customized and opened to meet particular needs. Adjusting dimensions, modifying layer height settings to ensure optimal printing quality, and aligning components to ensure proper assembly are all components of this customization. After the required adjustments have been implemented, the file is exported in a format that is compatible with the 3D printer that is being utilized for production. By assembling the component parts, the final casing for the tCam-Mini is created.

## 6 Operation instructions

The process of operating a hexacopter with a Flysky FS-i6X controller generally consists of the following steps:

• Inspect the controls: The user must acquaint themselves with the diverse controls of the Flysky FS-i6X controller. These consist of a pair of joysticks, with one controlling throttle and yaw, and the other controlling pitch and roll. Additionally, there are trim buttons, switches, and knobs.
• Throttle Calibration: Prior to flight, verify that the throttle stick is positioned at its minimum level to avoid unintended launch. Certain controllers have a throttle calibration procedure that establishes the minimum throttle position.
• Mode Selection: Adjust the controller settings based on the flight modes of the hexacopter. This entails adjusting switches or knobs to regulate flight modes such as stable mode, altitude hold, GPS mode, or manual mode.
• Arming: The hexacopter’s motors must be activated in order to get ready for flight. Typically, this is accomplished by manipulating a designated control (such as a throttle stick) to a predetermined position while simultaneously pressing a set of buttons or switches on the controller. During takeoff, the throttle must be gradually raised to elevate the hexacopter from the ground. Utilize the pitch and roll controls to maintain stability of the hexacopter while it ascends.
• Flight Control: The joysticks should be then used to manipulate the hexacopter’s motion. The left stick governs the throttle and yaw (rotation), while the right stick manages pitch and roll (forward/backward and side-to-side movement).
• Descent: When prepared to initiate the descent, steadily decrease the throttle in order to land the hexacopter. Utilize the pitch and roll controls to safely navigate it to the ground.
• Disarm: Upon landing, deactivate the motors of the hexacopter to avoid unintended motion. Adhere to the guidelines provided by the manufacturer to carry out the disarm operation.
• Power On: Make sure to activate both the hexacopter and the Flysky FS-i6X controller, ensuring they are turned on and correctly connected.

Strict adherence to safety protocols and regulations must be followed when operating a hexacopter. Refrain from flying in close proximity to individuals, structures, or any obstructions, and be aware of weather conditions that could impact the trip.

## 7 Validation and characterization

The integrated disaster-management system was validated through the deployment of a hexacopter and a FLIR Lepton 3.5 thermal camera, environmental sensors, and wireless communication capabilities. The platform was evaluated for its ability to perform aerial monitoring, environmental sensing, and real-time data transmission in disaster-response scenarios.

The integrated disaster-management system and the assembled hexacopter during field deployment are illustrated in Fig. 15 and Fig. 16, respectively.

**Fig. 15:**
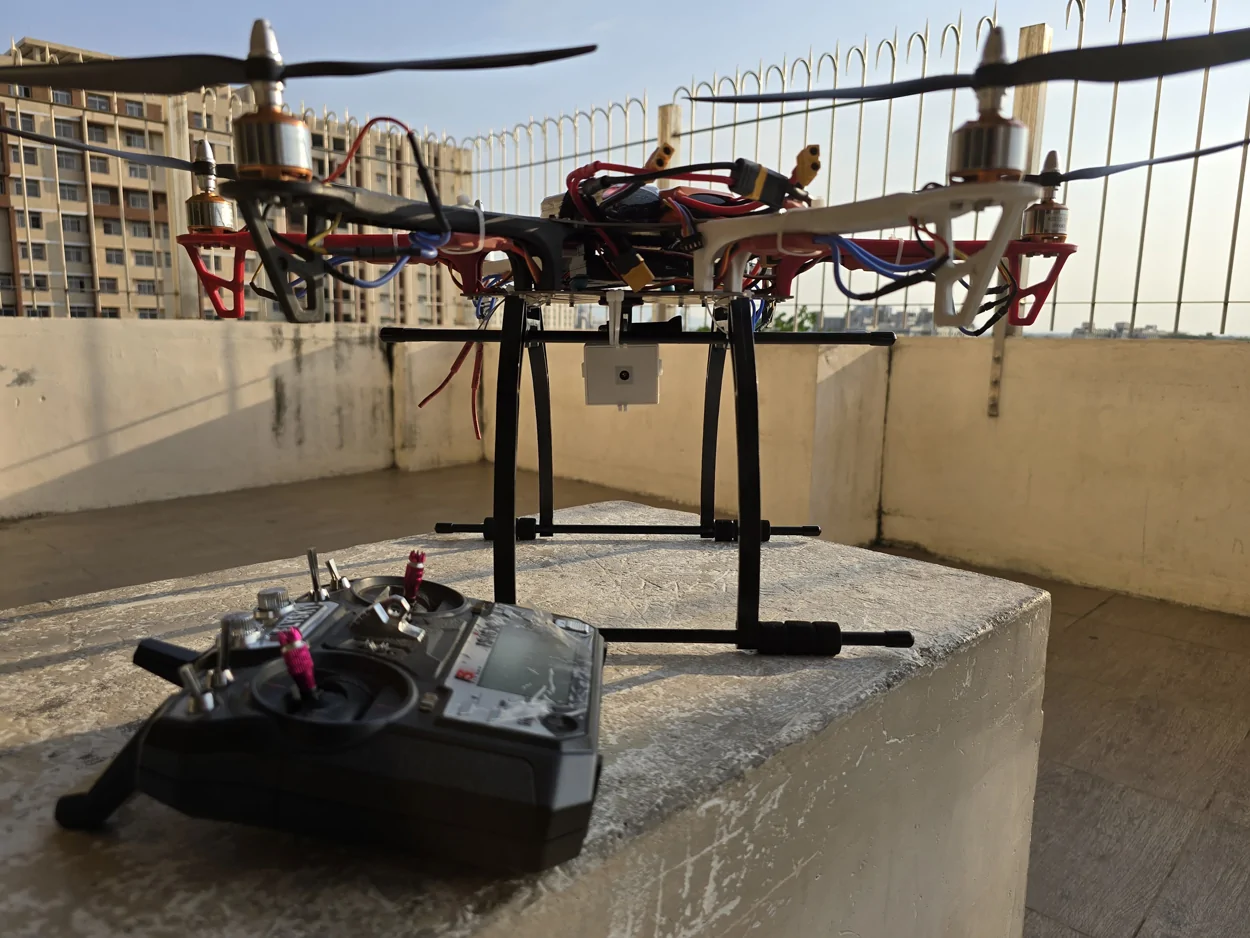
Integrated System.

**Fig. 16:**
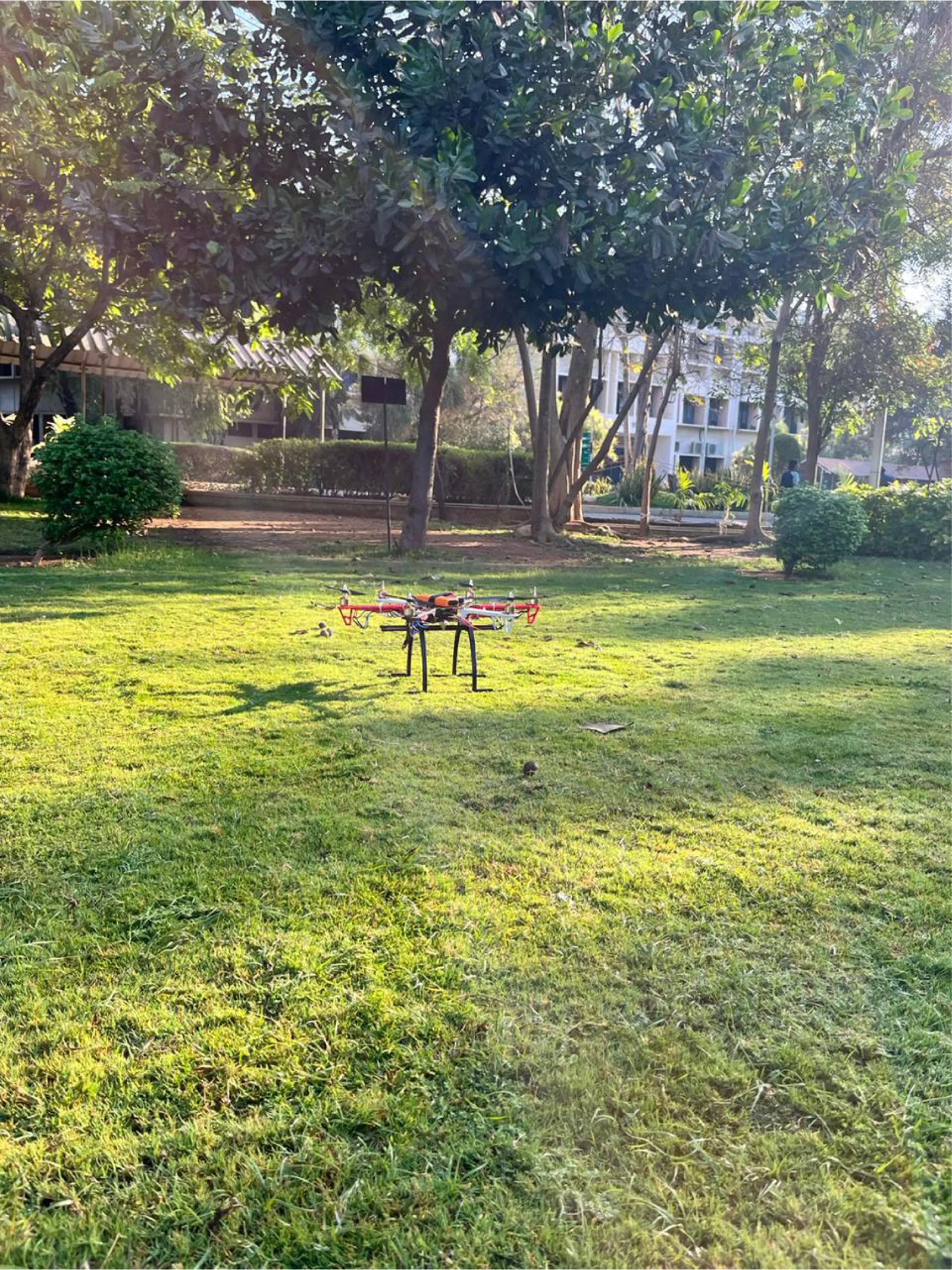
Assembled Hexacopter On-Field.

• The project utilizes a hexacopter drone integrated with an Arduino-based LiDAR system for accurate and continuous landscape mapping.
• The drone’s operations are currently controlled manually using a remote controller.
• The hexacopter platform supports additional functionalities, including a gripper mechanism for object retrieval and temperature and humidity sensors for environmental monitoring.
• The FLIR Lepton 3.5 thermal camera enhances the system’s capability to detect living organisms and thermal anomalies.
• Integration of an ESP32 communication module enables wireless transmission of thermal imaging and sensor data, allowing real-time monitoring via the internet.
• The platform enables rapid assessment and mapping of disaster-affected areas in real time.
• Integration with Geographic Information System (GIS) databases and disaster-response frameworks improves situational awareness and operational decision-making.
• Multi-sensor fusion combines LiDAR, thermal imaging, and environmental sensing data to provide a comprehensive understanding of the surrounding environment.
• The system architecture supports autonomous flight algorithms for navigation in disaster environments, reducing response times and minimizing human exposure to hazardous conditions.
• Communication relay functionality enables the establishment of temporary communication networks in areas where infrastructure has been disrupted.
• Data analysis and decision-support tools facilitate efficient interpretation of collected information and support effective resource allocation during disaster response operations.
• The modular design provides a foundation for continued research, development, and capacity building in drone-assisted disaster management.
• Future developments could potentially include the implementation of machine-learning techniques to enable autonomous and intelligent operation.
• Continuous research and development are crucial to enhance drone technology specifically tailored for disaster management.

### 7.1 Constraints

• As an undergraduate research project with limited funding, hardware selection was constrained by budget considerations. Nevertheless, the prototype successfully demonstrated the feasibility of the proposed system and its potential for further development.
• The Garmin LiDAR-Lite V3 sensor was limited to approximately 92,000 data points per complete scan, resulting in scanning durations that could extend over several hours.
• Reliable serial communication required transmitted data strings to maintain a fixed length, necessitating the addition of leading zeros to angular measurements and sensor values.
• The LiDAR sensor exhibited reduced measurement accuracy when scanning highly reflective surfaces and transparent materials such as glass, due to the inherent limitations of laser-based ranging technology.
• Although the Arduino-based LiDAR system successfully demonstrated three-dimensional terrain mapping, integration with the hexacopter was constrained by the high power consumption associated with continuous operation and real-time data processing.
• The FLIR Lepton 3.5 thermal camera and certain LiDAR technologies are subject to International Traffic in Arms Regulations (ITAR), requiring compliance with export-control requirements and restricting access to some technical documentation and support resources.
• Adherence to ITAR regulations imposed additional administrative and operational requirements, affecting project timelines, supplier selection, and collaboration opportunities.
• Sharing LiDAR-derived data and technical information with foreign entities required careful consideration to ensure compliance with applicable export-control regulations.
• The integration of LiDAR and thermal imaging sensors significantly increased the platform’s power requirements, creating challenges in balancing sensor operation, flight stability, and mission duration.
• Continuous LiDAR operation requires sustained laser emission and computational processing, while thermal imaging systems require uninterrupted power for image acquisition and analysis, making efficient power management a critical design consideration.

## CRediT authorship contribution statement

**Harshitha Vuucha:** Writing – original draft, Visualization, Investigation, Data curation. **Jeff Jomon:** Writing – review & editing, Supervision, Methodology, Formal analysis, Conceptualization. **Dheeraj Somasundaram:** Writing – review & editing, Validation, Software, Resources, Funding acquisition. **Monica Subashini M.:** Project administration.

## Ethics statements

The work does not use any human or animal subjects.

## Declaration of competing interest

The authors declare that they have no known competing financial interests or personal relationships that could have appeared to influence the work reported in this paper.
